# A contraindication to orthodontic and endodontic treatment: periapical cemento-osseous dysplasia

**DOI:** 10.1590/2177-6709.25.5.017-022.oin

**Published:** 2020

**Authors:** Alberto Consolaro, Omar Hadaya, Renata Bianco Consolaro

**Affiliations:** 1 Universidade de São Paulo, Faculdade de Odontologia de Bauru Bauru/SP, Brazil.; 2 Universidade de São Paulo, Faculdade de Odontologia de Ribeirão Preto Ribeirão Preto/SP, Brazil.; 3 Digital Center Radiologia Maringá/PR, Brazil.; 4 Faculdades Adamantinenses Integradas Adamantina/SP, Brazil.

**Keywords:** Periapical cemento-osseous dysplasia, Orthodontic contraindications, Endodontic contraindications, Pulp vitality, Tooth movement

## Abstract

**Introduction:**

The dental pulp is completely normal in teeth with periapical cemento-osseous dysplasia. However, orthodontic and endodontic treatments are contraindicated in cases with this injury.

**Objective:**

Present some biological, clinical and imaging reasons opposing these contraindications and questioning which are the real ones impediments and the reasons for the lack of research on the disease, analyzing cases submitted to orthopedic treatment under controlled and ethically approved conditions.

**Conclusion:**

The clinician can act safely based in available knowledge and aware of the possible consequences of orthodontic movement in teeth with periapical cemento-osseous dysplasia, as well as in the proper way of making a safe and definitive diagnosis.

## RESUMO

**Introdução:**

A polpa dentária é completamente normal nos dentes com displasia cemento-óssea periapical. Porém, os tratamentos ortodônticos e endodônticos estão contraindicados nos casos com essa lesão.

**Objetivo:**

Apresentar algumas razões biológicas, clínicas e imagiológicas, contrapondo-se essas contraindicações e questionando quais seriam os reais impedimentos e os motivos da falta de pesquisa sobre a doença, analisando casuísticas submetidas ao tratamento ortodôntico sob condições controladas e eticamente aprovadas.

**Conclusão:**

O clínico pode agir de forma segura embasado no conhecimento disponível e consciente das possíveis consequências da movimentação ortodôntica nos dentes com displasia cemento-óssea periapical, bem como na forma adequada de se fazer o diagnóstico seguro e definitivo da doença.

Orthodontists should, also, have a general knowledge of clinical practice, which includes recognizing normal structures and those that have small and large extra- and intraosseous lesions. 

 The term “lesion” means any permanent or transient anatomical change that requires a diagnosis. Orthodontist are not required to make an accurate clinical and imaging diagnosis, but they should be able to see that something is not right and seek the support of specialists in oral and maxillofacial lesions, such as stomatologists, oral pathologists and oral and maxillofacial surgeons.

In the few cases in which orthodontic treatment is contraindicated, very often the patient has inherent biological limitations, and there are no technical limitations of the orthodontic specialty.^1^ Patients should receive a detailed explanation, based on solid data and demonstrations of the consequences of orthodontic treatment if it is conducted in spite of these contraindications. 

In these cases, the communication of the orthodontist and the other specialist with the patient and his family should be repeated and explained thoroughly to avoid being affected by ignorance and equivocal information that may be received later from other people and professional, which may make patients and families feel insecure. Easy and immediate access should also be made available at this point so that they may talk about the insecurity and questions that may emerge when making these decisions.

Two clinical situations motivated us to present this paper to specialists in Orthodontics:

1) Cases of periapical cemento-osseous dysplasia in patients with endodontically treated teeth are not rare.
2) The frequency of patients with periapical cemento-osseous dysplasia undergoing orthodontic treatment has been growing. Imaging diagnoses of these cases are eventually made using periapical radiographs or CT scans to investigate why mandibular anterior teeth are not moving as expected during the progression of the treatment.

## WHY NOT MOVE TEETH ORTHODONTICALLY OR TREAT THEM ENDODONTICALLY IN CASES OF PERIAPICAL CEMENTO-OSSEOUS DYSPLASIA?

Teeth with periapical cemento-osseous dysplasia have a normal fully formed pulp and no inflammation or early ageing, let alone calcific metamorphosis, metaplasia of the pulp, or aseptic pulp necrosis.

In periapical cemento-osseous dysplasia, osteoblasts, clasts or both initiate the replacement of local bone with a cellularized connective tissue in the periapical region, which slowly produces a new mineralized tissue in the place, grossly similar to tooth cement. Its cause remains unknown and, although it is expected to originate in a genetic abnormality, the chromosome and gene affected have not been determined. There is no biological explanation to why it occurs so often and is limited to the alveolar bone in this region.

Mineralized tissue produced by newly formed cellularized connective tissue in cases of periapical cemento-osseous dysplasia is disorganized, little mineralized and does not reorganize the region so that the alveolar periapical structures are reconstructed, and leaves them without a lamina dura or trabecular bone and, especially, no periodontal ligament.

As self-limiting bone resorption and replacement of an average of 1 cm in diameter from the tooth apex, newly formed cellular fibrous tissue does not take up pulp space or compress the vessels that supply the dental pulp. In other words, there is no inflammation or pulp necrosis. 

**Figure 1:**
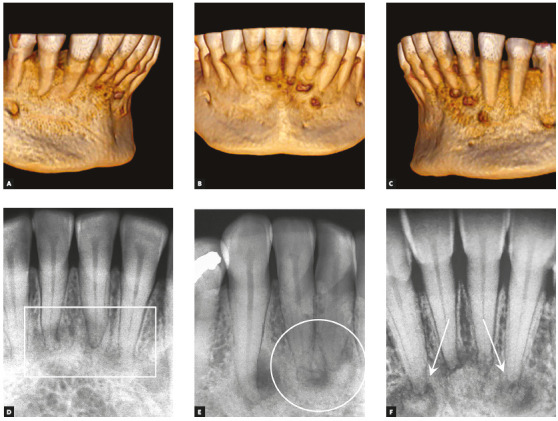
A, B, C) 3D reconstruction of CT sections of mandibular incisors and canines in patient with intermediate-stage periapical cemento-osseous dysplasia. First periapical radiograph (D) of another patient at initial stage: newly-formed mineralized and cementoid tissue has not been deposited or mineralized. Second image (E) shows denser mineralized areas still separated from apex (circle). Third image (F) shows mineralized tissue randomly continuous with apex (arrows).

**Figure 2:**
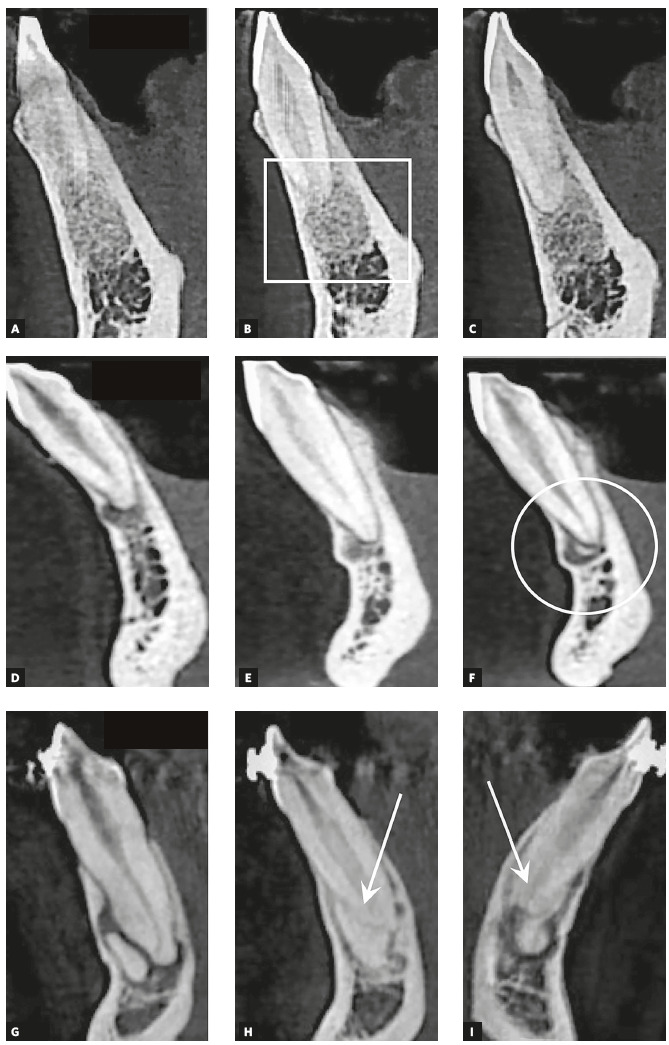
CT sections (lateral/sagittal view) of mandibular incisors of three patients with periapical cemento-osseous dysplasia. In first group of images (A, B, C), disease is at initial stage; newly-formed mineralized and cementoid tissue has not been deposited or mineralized, which generates irregular hypodense area mottled with delicate hyperdense dots (box). In second group (D, E, F), densely mineralized area is still separated from apex (circle). In third group (G, H, I), mineralized tissue fuses with tooth and is randomly continuous with tooth structure (arrows).

As it fills the periapical space, the newly mineralized tissue gradually approximates normal cement and merge with it, displacing cementoblasts to neighboring areas, establishing continuity with the tooth (Figs 1 and 2). A precise detection of what was a lesion and what was a tooth becomes impossible in the intermediate and advanced phases of periapical cemento-osseous dysplasia.

Resorption “respects” and does not involve apical dental tissues, only the tissues of the periodontal ligament and bone (Figs 1 and 2). The reason why these dental structures are preserved is unknown, but tissue section under microscopy do not show any local cementoblast or odontoblast necrosis. These cells - cementoblasts and odontoblasts - do not have receptors for bone remodeling mediators, and are not genetically anomalous in the process in which only periapical alveolar osteoblasts are involved.

Along time, there is no replacement of apical dental tissues with newly formed tissue produced during periapical cemento-osseous dysplasia, which would characterize replacement resorption. Newly formed tissue combines or continues the tooth structures, but with no tooth resorption or replacement.

Orthodontic treatments should not be conducted because, once periapical cemento-osseous dysplasia is established, cellularized connective tissue replaces the periodontal ligament and produces islands and trabeculae of newly formed mineralized tissue, very similar to cementum, but disorganized. The essential tissue for tooth movement in orthodontic treatment is the periodontal ligament,^1^ where osteoclasts, new cementoblasts and osteoblasts circulate. This ensures that, after each appliance activation, alveolar tissues, including apical cementum, ligament and alveolar bone reorganize and prepare for a new movement cycle to start immediately after that.

The newly mineralized tissue thus produced unites with the tooth, and, as there is no periodontal ligament, tooth movement is impossible. However, we may ask ourselves: before any mineralized tissue is seen on CT scans and radiographs, would it be possible to move teeth that have periapical cemento-osseous dysplasia lesions that still appear fully radiolucent? 

We may say that, yes, it is physically possible, but tooth movement is not a physical event, but, rather, a biological event mediated by cells and chemicals.^1^ Minor movements may be possible, but not to achieve substantial changes in the position of the teeth involved. If periapical cemento-osseous dysplasia is at its intermediate or final stage, mineralized tissue will already be united and fused with the tooth, and tooth movements will be impossible.

In sciences, including biology and medicine, there are no moments in which we should use the words “always” or “never”! Even in the initial stage, and still in the intermediate and final stages, there are no reports of cases of application of orthodontic forces to teeth with periapical cemento-osseous dysplasia in controlled and ethically approved study protocols to evaluate this treatment alternative.

## ASPECTS AND TREATMENT OF PERIAPICAL CEMENTO-OSSEOUS DYSPLASIA

1) Periapical cemento-osseous dysplasia is not a neoplasia; it is, rather, characterized by a pseudotumoral fibro-osseous lesion without a history of becoming malignant^2^. However, some authors^2^ found that it may be an initial form of florid cemento-osseous dysplasia. In these cases, the risk of this condition should be evaluated thoroughly, and one way to do this is to carefully examine all the mandibular bone to detect initial radiolucent areas.
2) A diagnosis of periapical cemento-osseous dysplasia is more common in black women of about 40 years of age, and this should be taken into consideration. In younger patients, cases are usually diagnosed at its initial, or radiolucent, stage, and it may affect one or more incisors too! In this stage and condition, patients may equivocally receive a wrong diagnosis and an indication of endodontic treatment, even though pulp vitality is detected. An endodontic treatment may lead to contamination of the area and extravasation of material without any beneficial effects for the progression of the disease, and should, therefore, be avoided in all cases.
3) If the teeth affected by this disease also have caries or periodontal disease, they should be treated as usual for these conditions, considering that the pulp is vital and only the periodontal ligament is affected. If there is pulp necrosis for any other reason, more often because of advanced caries or, also, dental trauma, the endodontic treatment is necessary and should follow the usual criteria for it.
4) Some possibilities are raised - and maybe even adopted - such as endodontic surgery including the apex of all incisors, removed using curettage, or the full removal of apical thirds and periapical alveolar bone, as the lesion is encapsulated. There are no cases in the literature that describe the benefits a patient would have, but if endodontic surgery repairs the lesion, would it be possible to move teeth orthodontically? In theory, considering what we know about the orthodontic treatment of teeth that underwent endodontic surgery, but before we do it, controlled and ethically approved studies should be conducted to evaluate this option for the treatment of periapical cemento-osseous dysplasia.

## WHAT WOULD BE THE CONSEQUENCES OF MOVING TEETH WITH PERIAPICAL CEMENTO-OSSEOUS DYSPLASIA?

1) Will there be more tooth resorptions in these patients? We do not know that, but the distribution of forces on the roots and alveolar bone becomes random and out of the control of the orthodontist.
2) Is there any risk of pulp necrosis in these teeth? Probably no, but there are no data to confirm this hypothesis.
3) Is there any, no matter how slight, chance of this type of lesion becoming malignant? No, periapical cemento-osseous dysplasia is a change of cell function, and not a cell proliferation disorder.
4) Is there any chance of microbial contamination in the area? Also no, because the biological process of moving teeth does not contaminates tooth tissues.
5) Is there more pain and discomfort for the patient during orthodontic treatment? Maybe yes, because the biological process of movement becomes random and may generate mediators of inflammation and pain!
6) Are buccal and lingual bone losses probable? It is impossible to predict that, but tooth movement, as well as the distribution of forces, becomes random.
7) Would there be a greater chance of periapical cemento-osseous dysplasia progressing into florid cemento-osseous dysplasia because of tooth movements? It is impossible to know without any studies about it.

## HOW TO MAKE A SAFE AND DEFINITIVE DIAGNOSIS OF PERIAPICAL CEMENTO-OSSEOUS DYSPLASIA?

Tooth and periodontal changes should not be diagnosed based on panoramic radiographs, because they have several distortions and superimpositions that may lead to false positive or false negative results. Even more important is that this diagnosis of dental and periodontal changes should not be made in the anterior area of the maxilla and mandible. 

Several cases of periapical cemento-osseous dysplasia are not visualized on panoramic radiograph, particularly, in its initial stage, when the lesion is still radiolucent This disease affects mandibular incisors in particular, and may extend to canines and, rarely, to premolars. 

In several cases, periapical cemento-osseous dysplasia is diagnosed during orthodontic treatment, because it began before the ideal conditions had been ensured: periapical radiographs of all teeth obtained before and after orthodontic treatment. If only panoramic radiographs are obtained for planning and treating orthodontic cases, the diagnosis of some changes, such as tooth resorptions, caries and periodontal disease, may be missed.

Periapical cemento-osseous dysplasia during orthodontic treatment planned without periapical radiographs are eventually diagnosed because the anterior teeth do not move, in spite of the application of forces. To investigate it, CT scans or periapical radiographs are obtained when periapical cemento-osseous dysplasia is detected, now already in its intermediate stage.

The diagnosis of periapical cemento-osseous dysplasia is made based on clinical and imaging findings, and microscopic evaluations are not necessary, as the images and signs and symptoms are unique and characteristic of this disease, ensuring a safe diagnosis. No biopsy is necessary.^2^ CT scans provide a reconstruction and three-dimensional assessment of the condition at a certain moment (Figs 1 and 2). As years go by, the mass of irregular mineralized tissue is hyperdense, if continuous to teeth, are separated from bone by an irregular and imprecise hypodense or radiolucent halo on radiographs.

Another factor to be taken into account in the diagnosis of periapical cemento-osseous dysplasia is the patient’s profile, as most are black women in their forties. Ethnicity interacts closely with periapical cemento-osseous dysplasia, a disease that probably results from a genetic defect that induces it. In Brazil, 75% of the people have genes of this ethnicity, as the level of mixed ethnicity is high among the Brazilian population. Therefore, patients, even men, that seem to be white and have a Caucasian profile may present with this disease.

## FINAL CONSIDERATIONS

Before controlled, ethically approved studies with patients with periapical cemento-osseous dysplasia are conducted, these patients should not undergo orthodontic treatments, as safe results and precise risk of damages cannot be predicted. Individual clinical attempts to treat these patients orthodontically may be laudable in that it is a sign of the desire to do it right for our patients. However, they may be ethically and legally questionable, as there is no scientific basis derived from methods applied in controlled studies including series of cases of periapical cemento-osseous dysplasia.
